# Supplementary material for: Are carbon emissions trading and green financial instruments synergistic? -Comprehensive quantitative research based on content analysis
Source: PLoS One. 2024 Mar 7;19(3):e0298601. doi: 10.1371/journal.pone.0298601 (PMC10919601; doi:10.1371/journal.pone.0298601)
Supplement: S1 Data — (ZIP) [file pone.0298601.s001.zip › Data/地级市Z_F工作报告.zip!/╜¡╦╒/─╧╛⌐╩╨/└┤╫╘╣1⁄2╓┌║┼í╛┬φ┐╦╩2╛▌═°í┐_2019.docx]

各位代表：

现在，我代表南京市人民政府，向大会报告工作，请予审议，并请市政协各位委员和列席人员提出意见。

一、2018年工作回顾

2018年是全面贯彻党的十九大精神开局之年，也是本届政府履行职责的第一年。面对错综复杂的宏观环境和艰巨繁重的改革发展任务，我们坚持以习近平新时代中国特色社会主义思想为指导，认真落实党中央国务院、省委省政府决策部署以及市委工作要求，坚持稳中求进工作总基调，坚持新发展理念，按照高质量发展要求，对标找差、创新实干，统筹推进稳增长、促改革、调结构、惠民生、防风险各项工作，圆满完成十六届人大一次会议确定的目标任务，“强富美高”新南京建设迈出新步伐。

——经济发展呈现良好势头。预计全年完成地区生产总值1.27万亿元左右，可比价增长8%；一般公共预算收入1470亿元，同口径增长11.1%，两项指标增速均快于全省、高于全国。同时，规上工业增加值等指标增幅创近年来新高；一般公共预算收入总量、增幅等指标在GDP万亿元城市中实现进位。

——创新名城建设精彩开局。全年净增高新技术企业1282家，增长近70%，增量和增幅均位居全省第一；高新技术产业产值增长17%，首次突破万亿元大关；新增就业参保大学生34万人，增长60%；南京高新区在全国排名提升7位；发明专利授权量位居全省第一。南京着力打造集聚创新资源“强磁场”经验获国务院通报表扬。

——重点领域改革亮点纷呈。工程建设项目审批制度“864”改革处于全国第一方阵；“不见面审批”“预审代办制”等“南京经验”在全国推广；不动产登记效率在国务院第五次大督查营商环境评级中位居全国第一。以“客户需求导向、国际惯例标准、落实改革要求”为特点的全国最优营商环境示范城市创建全面启动，在《中国城市营商环境报告2018》中南京跻身全国前十。

——交通枢纽地位加快提升。南京铁路枢纽总图规划正式获批，以南京为中心、辐射全国全省的“米”字型高铁网络进入建设实施阶段。禄口机场加快建设全国服务最佳机场，高铁、机场换乘服务体系建成投运。重大交通基础设施建设大力度展开，7条轨道交通线路、6条过江通道同步建设，全市地铁通车里程达到378公里，位居全国第四位。

——年度民生实事全面完成。公共财政支出75%用于民生保障，35件民生实事兑现到位，社区养老、加装电梯等工作走在全国全省前列。南京在全国最有安全感十大城市中位列第四，连续10年被评为中国最具幸福感城市。

回顾过去的一年，困难多于预想，结果好于预期。我们重点抓了以下几项工作：

一是积极应对宏观经济形势变化，经济实现持续健康发展。面对经济下行压力，我们加强组织、有效应对，经济运行总体平稳、稳中有进、进中提质。扎实做好“六稳”工作。大力促进就业创业，城镇新增就业30万人，创历史新高；加强政府债务风险防控，着力打击非法集资等非法金融活动，及时处置企业资金链风险，坚决守住不发生系统性金融风险底线；全力推动外贸稳增长，预计实现进出口665亿美元，增长8.7%；全面落实扩大外商投资政策举措，实际利用外资38亿美元，增长5%；着力扩大有效投资，固定资产投资增长9.5%，其中工业投资增长8.8%，比上年提高7.8个百分点，484个省市重大项目进展顺利；金融机构本外币存贷款余额分别增长10.9%、14.8%。着力提升质量效益。落实高质量发展指标体系和考核办法，高质量发展导向进一步强化。服务业增加值占地区生产总值比重达60.7%；规上工业增加值增长8%，创两年来新高，战略性新兴产业占工业增加值比重达到42.5%。完成社会消费品零售总额5846亿元。新增上市企业7家，其中新增境内上市公司数量位居全国第四；11家民营企业进入全国500强。稳步推进“三去一降一补”。关停化工企业、整治铸造企业各42家，“十三五”船舶去产能任务全面完成。梅钢产能布局调整取得重要进展。房地产市场保持平稳健康发展，租购并举制度加快建立。

二是深入实施创新驱动发展“121”战略，创新动能加速集聚。把创新驱动发展作为核心战略，举全市之力建设具有全球影响力的创新名城。创新名城布局全面展开。制定出台“1+45”政策体系，全力推进“两落地一融合”等十大工程。将83个科创载体整合为15个高新园区，并依法赋予165项审批权限，打造创新名城建设主阵地。科技产业创新中心建设成效明显。集中签约新型研发机构208个，孵化引进企业951家，新增3名诺贝尔奖得主、55名国内外院士来宁创新创业，斯坦福、剑桥等一批国际一流高校创新团队落户南京。新增科创型企业2.4万家、增长17%。培育集聚高成长性科技企业150家，首批29名高层次人才通过“举荐制”产生。综合性科学中心启动建设。网络通信与安全紫金山实验室正式揭牌，未来网络试验设施获得国家发改委批复，列入国家“十三五”重大科技基础设施指南的作物表型组学研究、生物医学大数据等项目加速培育。《自然》杂志最新发布全球科研城市50强榜单，南京位列第12位。创新生态加快营造。出台“股权投资十条”，组建注册资本50亿元的创新投资集团。与深交所合作建设“南京科技金融路演中心”，与上交所共建南京资本市场学院，全市股权投资基金管理规模同比增长30%。成功获批国家知识产权运营服务体系建设重点城市，中国（南京）知识产权保护中心正式运行。

三是着力打造产业特色和提升产业高度，转型升级迈出坚实步伐。围绕“4+4+1”主导产业方向，进一步聚焦重点产业、特色产业，抢占产业发展制高点，产业结构迈向中高端。推动制造业高质量发展。坚持工业兴市，四大先进制造业产值增长16%左右。启动产业地标打造，研究制定集成电路和新能源汽车两个产业地标行动计划。集成电路产业集聚上下游企业200余家、产值增长55%，台积电项目实现量产；新能源汽车产量接近10万辆，实现产值300亿元，增长150%。推进“两化”深度融合，未来网络研究院、中电熊猫入选工信部工业互联网创新发展工程支持计划。工业技改投资占工业投资比重超过61%。推动现代服务业转型提升。促进服务业集聚区提档升级，新增3家省级生产性服务业集聚示范区。苏宁易购、运满满等互联网平台企业规模壮大，阿里巴巴江苏总部、小米华东总部相继落户。四大现代服务业主营业务收入增长12%，软件业务收入达到4400亿元，旅游业总收入增长13.4%，金融业增加值占GDP比重预计达到10.5%，文化产业增加值占GDP比重预计达到6.3%。推动现代农业高效发展。农业现代化水平保持全省首位，都市现代农业发展水平位居全国第三。南京国家现代农业产业科创园展示中心建成。南京农业嘉年华入选首届“中国农民丰收节”活动。

四是统筹推进规划建设管理，城乡功能品质显著提升。深入贯彻中央城市工作会议精神，聚力提升省会城市功能和中心城市首位度，打造更加宜创宜居宜业城市。城市空间布局持续优化。高标准推进新一轮城市总体规划修编，深入实施“多规合一”，城市设计、城市双修“双试点”取得阶段性成果。江北新区直管区基本实现控制性详规全覆盖，中央商务区、研创园等片区加快建设，国际健康城确立“三中心一高地”发展新定位。河西新城、南部新城开发持续推进。综合交通体系加快构建。南沿江城际铁路正式开工，长江五桥等重大基础设施进展顺利，长江大桥公路桥维修改造工程竣工通车，地铁宁溧线建成运营，红山路—和燕路、扬子江大道节点改造有序推进，打通“断头路”专项行动加快实施。长江南京以下12.5米深水航道二期工程建成试运行，南京至长江出海口深水航道全线贯通。城市更新改造有序推进。颐和路等历史文化街区保护利用不断加强。新开工保障房456万平方米，竣工356万平方米；改造棚户区488万平方米。实施8个重点片区环境综合整治、16条主次干道及沿街建筑立面出新，完成192个老旧小区、600条背街小巷整治。乡村振兴战略有力实施。建成美丽乡村示范村80个、宜居村100个，打造6个市级田园综合体。完成农村公路提档升级452公里、农路桥梁20座。江宁区成功创建“四好农村路”全国示范区。精细化管理不断深化。深入实施精细化建设管理十项行动，城市功能面貌加快提升。实施玄武湖、秦淮河夜景亮化工程，“最南京”夜景名片形象初显。开展交通堵点治理攻坚，新建成65个公共停车场，新增停车泊位9万多个。在550个小区推进垃圾分类，覆盖居民40余万户。

五是聚力打好污染防治攻坚战，生态文明建设取得明显成效。坚持问题导向，狠抓重点关键，推动环境质量持续改善。长江大保护扎实推进。出台《长江岸线保护办法》，在全国率先把长江岸线保护纳入法治化轨道。把修复长江生态环境摆在压倒性位置，调整优化岸线规划，长江岸线开发利用率下降到50%以下。环境质量明显提升。坚决抓好中央环保督察“回头看”问题整改，持续开展“263”专项行动，解决了一批突出问题。坚持科学治水、系统治水、精准治水、高效治水，提标改造城北、江心洲等污水处理厂，建成区基本消除黑臭水体，22个省考以上断面全部达标，完成雨污分流片区年度改造任务；5个主要入江支流控制断面消除劣V类。完成204家重点企业挥发性有机物专项治理，建成143家“智慧工地”，初步监测统计空气优良率为70.4%。土壤污染管控和修复不断强化。节能减排成效显著。万元GDP能耗完成省定目标，两年累计削减非电用煤280万吨左右。出台实施工业企业资源集约利用绩效综合评价差别化政策指导意见，倒逼企业进一步节能降耗、提质增效，全市建设用地亩均GDP达43.5万元。盘活2.76万亩城镇存量用地。生态建设加速推进。完成绿化造林3.85万亩，林木覆盖率达31.02%，建成区绿化覆盖率达40.75%。新改建游园绿地22个，公园绿地服务半径覆盖率超过92%。

六是持续深化改革开放，发展动力活力进一步增强。全力推进重点改革，持续扩大对外开放，取得了明显成效。重点领域改革进展顺利。持续推进“放管服”改革，进一步简政放权，取消21项、承接35项行政权力事项。不断深化“3550”改革，积极拓展“不见面审批”服务，获选全国“企业开办全程网上办”改革试点，新登记各类市场主体21.6万户。全面完成市、区（园区）、街镇、村居四级政务服务体系建设。国资国企改革深入推进，市旅游集团被列入国家“双百行动”改革试点。内贸流通体制改革持续深化，南京入选全国供应链“双试点”城市。财政综合管理和PPP两项工作获国务院“真抓实干”表彰。江宁集成改革试点全面启动，农村综合改革、户籍制度改革、公立医院改革等各项改革稳步推进，价格、审计、统计工作得到加强。对外开放步伐加快。坚持“引进来”和“走出去”并举，对外投资42亿美元，居全省首位。推进国家服务贸易创新发展试点，服务贸易进出口151.2亿美元，增长12%。江宁开发区和南京开发区综合排名分别提升至全国第8、第11位，国家跨境电子商务综合试验区正式获批。口岸整体通关效率提升33%，集装箱进出口合规成本降低100美元，完成货物吞吐2.52亿吨、集装箱320万标箱。全年开行中欧班列142列、6495车。禄口机场年出入境旅客增长16.29%，国际货邮吞吐量增长14.5%。国际化程度不断提升。成功举办世界智能制造大会、世界名城会、2018市长国际咨询会议等重大活动；圆满举行羽毛球世锦赛、南京马拉松等10项国际大赛，体育城市指数位列全球第十；成为国内首个国际和平城市。区域协作深入推进。加快推动南京都市圈协同发展、宁镇扬同城化发展，在基础设施互联互通、环境联防联控、市场共建共享等方面取得新成效。宁句城际正式开工，宁宣、宁合、宁马等高速公路新建扩建工程全面实施。扎实做好对口支援和扶贫协作工作，落实援助资金8.5亿元。

七是全力突破市场主体关心关注的问题，营商环境加速优化提升。制定出台优化营商环境100条、支持民营经济健康发展30条，推出了一批首创和领先举措，全力打造全国最优营商环境示范城市。企业税费负担切实减轻。积极实施小微企业、科技型初创企业普惠性税收免除等政策，全年涉企减税591.8亿元、出口退税209亿元。清理精简行政审批事项和涉企收费，降低企业用电、用地、制度性交易成本，涉企减费23.6亿元。稳定社保缴费方式，落实困难企业缓缴社保费政策，减轻企业负担45亿元。民企融资难题有效化解。设立民营企业纾困与发展基金、民营企业转贷互助基金，全力打出支持民营经济、服务中小微企业发展融资的“组合拳”。完善金融机构信贷激励机制，激励商业银行加大对民营企业贷款投放力度。做大做强新兴产业发展基金，19支子基金正在加快组建。服务企业方式不断创新。积极推进“互联网+政务服务”建设，创新审批服务方式。举办新产品新产业新应用“三新”推介对接会，加大政府采购支持民营企业力度。积极开展“支持实体经济发展主题服务月”活动，走访1.64万家企业，解决企业诉求7000多件。

八是切实保障和改善民生，市民群众获得感不断增强。践行以人民为中心的发展思想，下大力气解决好市民群众关心关注的热点问题。居民收入实现新增长。全体居民人均可支配收入增长9%，快于经济增长。坚持精准扶贫、精准脱贫，符合市定标准的低收入农户和经济薄弱村脱贫率分别达到96%、96.5%。保障水平得到新提升。低保标准提高到每月860元，残疾人基本保障和重点救助实现全覆盖；企业退休人员月均养老金达2984元，位居全省第一；医疗救助标准不断提高。民生事业获得新发展。普通高中增加招生计划4500人，“弹性离校”“空中课堂”等做法得到教育部肯定，省教育现代化监测得分实现“三连冠”。市中医院新院、鼓楼医院江北国际医院建成运营，基层医疗机构医联体实现全覆盖。每千名老人拥有养老床位数达37.7张，新增社区老年人助餐点250个，获评全国居家和社区养老改革试点优秀地区。公共文化服务设施面积达210万平方米。社会治理得到新加强。成立全国首家网格学院，社区治理和服务创新实验区数量位居全国第一。扎实开展大排查大整治行动和扫黑除恶专项斗争，严格落实安全生产责任制，安全生产、食品药品、信访形势稳定向好，社会大局和谐稳定。智慧城市建设取得积极进展，信用示范城市建设不断深化，质量工作得到加强，军民融合发展水平不断提升，法治政府建设扎实推进。同时，民族、宗教、档案、史志、社科、参事工作取得新进展，妇女、儿童、科普、老龄、仲裁、青少年、残疾人、红十字、慈善事业取得新业绩，外事工作、对台事务、港澳工作、侨务工作取得新成效。

过去一年，我们树牢“四个意识”，坚定“四个自信”，坚决做到“两个维护”，自觉扛起管党治党的政治责任。深入学习贯彻习近平新时代中国特色社会主义思想，扎实推进“两学一做”学习教育常态化制度化，积极开展解放思想大讨论和“推动高质量发展走在前列”大调研活动。坚持从严从实，突出抓好中央巡视反馈意见整改。认真贯彻落实中央八项规定及其实施细则，坚持不懈整治“四风”。严格执行国务院“约法三章”，市级“三公”经费支出同比下降3%。切实加强行政监督和审计监督，坚决查处和纠正违法违规行为，着力营造风清气正发展环境。自觉接受人大法律监督和政协民主监督，办理市人大代表建议311件、人大代表议案6件、政协委员提案735件，提请市人大常委会审议地方性法规草案6件，制定修改政府规章6件。

各位代表：历经天华成此景，人间万事出艰辛。过去一年取得的成绩，是以习近平同志为核心的党中央统揽全局、科学引领的结果，是省委省政府和市委坚强领导、科学决策的结果，是全市上下克难攻坚、团结奋斗的结果。在此，我代表市政府，向全市人民致以崇高的敬意！向各位人大代表、政协委员，向各民主党派、工商联、无党派人士，向各人民团体、各界人士和老同志，表示衷心的感谢！向关心支持南京建设的人民解放军和武警驻宁部队、中央和省驻宁机构单位、港澳台同胞、海外侨胞和国际友人，表示衷心的感谢！

在总结成绩的同时，我们也清醒地看到存在的差距和不足。主要表现为：省会城市功能作用发挥不够，中心城市首位度有待提升；工业经济不强，开放型经济发展有待加快，民营经济活力尚未充分激发，规模企业较少；科教资源优势有待进一步释放，交通枢纽功能有待提升；城乡区域发展不够平衡，优质公共资源配置不够均衡；对特大城市治理规律的把握、城市发展中“城市病”的化解还要进一步加强；生态环境与市民期待还有一定差距，社会治理和安全生产存在薄弱环节，部分领域风险隐患不容忽视；政府职能需要进一步转变，一些干部改革创新意识、担当负责精神还需提升，全面从严治党任务依然艰巨。对此，我们将增强忧患意识，坚持问题导向，针对性加以解决。

二、2019年总体要求和主要任务

今年是新中国成立70周年，也是决胜高水平全面建成小康社会的关键之年。做好今年的工作，形势特殊、责任重大、意义深远。应对外部环境的深刻变化，我们必须保持定力、精准发力，全力夯实“稳”的基础。当今世界正面临着百年未有之大变局，“变化”与“变革”的特征愈加明显；我国经济正在由高速增长加快转向高质量发展阶段，危和机同生并存。面对前所未有的复杂形势，全市上下要深刻认识到，克服了危即是机，失去了机即是危，我们必须定下心、稳住神、吃住劲，化危为机、危中寻机、以稳促进，聚精会神做好“稳就业、稳金融、稳外贸、稳外资、稳投资、稳预期”各项工作，围绕“巩固、增强、提升、畅通”八字方针下功夫，深化供给侧结构性改革，全力稳住经济社会发展好形势。实现高点定位的奋斗目标，我们必须抢抓机遇、聚焦重点，持续增强“进”的势头。时代因奋斗而常新，城市因梦想而行远。面对建设“创新名城、美丽古都”的发展愿景，面对提升省会城市功能和中心城市首位度的历史重任，我们必须只争朝夕、毫不松懈，一往无前、击鼓奋进，积极抢抓“一带一路”、长江经济带、长三角一体化等国家战略叠加交汇的重大机遇，落实“六个高质量”要求，聚焦建设创新名城、打造产业地标、优化营商环境、建设江北新区等事关长远发展的大事要事，更大决心、更大勇气、更大手笔、更大力度地谋发展、求突破、创一流，以实实在在的工作成效，推动南京各项工作走在全国全省前列。回应市民群众日益增长的美好生活需要，我们必须以民为本、牢记使命，不断创造“好”的生活。民之所望，施政所向。面对市民群众的殷切期待，我们要始终坚持以人民为中心的工作导向，通过有效的制度安排和扎实的工作举措，切实强化民生工作的稳定性、连续性、积累性，促进经济发展与民生改善有效互动、良性循环，让南京的发展更有“温度”，让南京高水平全面小康有更广代表性、更多获得感、更高满意度。

新的一年，我们要以习近平新时代中国特色社会主义思想为引领，全面贯彻党的十九大精神，认真落实习近平总书记重要指示和中央决策部署，按照中央经济工作会议以及省委十三届五次全会、省委常委会专题研究南京工作和市委十四届七次全会提出的要求，统筹推进“五位一体”总体布局，协调推进“四个全面”战略布局，坚持稳中求进工作总基调，坚持新发展理念，坚持推进高质量发展，坚持以供给侧结构性改革为主线，坚持深化市场化改革、扩大高水平开放，牢牢把握“创新名城、美丽古都”的发展愿景，牢牢把握提升省会城市功能和中心城市首位度的主攻方向，进一步解放思想、对标找差、创新实干，坚决打好“三大攻坚战”，全力做好稳增长、促改革、调结构、惠民生、防风险、保稳定各项工作，以推动高水平全面建成小康社会、高质量建设“强富美高”新南京的优异成绩庆祝新中国成立70周年。今年经济社会发展的预期性指标主要是：地区生产总值增长7.5%—8%；一般公共预算收入增长8%；固定资产投资增长8%，工业投资增长10%，高新技术产业投资增长12%；社会消费品零售总额增长9%；外贸进出口增长5%，实际利用外资40亿美元；全社会研发投入占GDP比重达到3.1%；全体居民人均可支配收入增长8%；城镇调查失业率和登记失业率控制在省定标准以内；居民消费价格指数控制在省定目标以内。约束性指标主要是：节能减排和大气、水环境质量确保完成国家和省下达的目标任务。

实现今年的目标，我们将重点做好十个方面工作：

（一）抢抓机遇，提升省会城市功能和中心城市首位度。以国家和省全力支持南京建设发展为动力，理直气壮讲首位度、全力以赴干首位度、名副其实增首位度。

推动经济发展稳中有进。坚持量质并举，全力推进经济高质量发展，力争经济总量规模与对标城市缩小差距，主要经济指标增速保持全省前列，在高质量发展监测考核中走在全省前列。把发展的着力点放在实体经济上，加快建设现代化经济体系。把消费对经济的拉动放在更加突出的位置，进一步扩大并优化商品和服务供给，满足最终需求，拓展市场新空间；改善和优化消费环境，完善消费领域基础设施和信用体系，引导实体商业转型升级和流通方式创新，加快培育新型消费业态和消费增长点；争取国际消费中心城市试点，实施商圈消费引领工程，着力构建智慧商圈、智慧商店、智慧物流，提档升级新街口商圈，改造建设湖南路商圈，推动夫子庙全国步行街改造提升试点，发挥“南京购物”对周边城市的辐射带动效应。

加快构建“米”字型交通网络。以推动对外大交通体系建设为重点，积极争创国家综合交通枢纽示范城市。积极推进南沿江铁路、南京北站客运枢纽建设，力争开工建设北沿江铁路、宁淮城际铁路和宁芜铁路外绕工程。大力完善高速路网，新建溧高高速、宁宣高速，扩建宁合高速、宁马高速等重点项目，畅通与都市圈城市之间的交通联系。建成龙潭疏港公路、328国道雍庄至龙池段等干线公路，构建完善市域高速、干线路网格局。

完善省会城市服务功能。强化金融功能，加快建设以江北新区和河西新城“一江两岸”为核心的高端金融服务集聚区，吸引更多金融机构落户，建设金融中心。提升商务功能，实施总部企业招引专项行动，突出打造江北新区、河西新城、南部新城等一批总部经济集聚区，提升总部经济集聚度。做强会展功能，建设空港滨水会展小镇，争取更多辐射面广、影响力大的展会落户南京，提升会展经济活跃度。推动现代服务业高端化发展，切实提高设计、健康服务、物流、中介服务等产业辐射度。推动枢纽经济发展，打造一批改革开放新平台。

发挥区域辐射带动作用。抢抓长三角一体化发展战略上升为国家战略的重大机遇，着力推进一批重大基础设施建设、争取一批国家政策资源、统一一批城市治理标准、建设一批共建共享项目，努力成为促进长三角一体化发展的战略支点、驱动长三角高质量发展的创新引擎、支撑长三角国际化发展的门户枢纽，提升南京在长三角的中心度。积极争取南京都市圈纳入国家布局，提升南京在苏皖两省都市圈城市中的辐射度。深入推进宁镇扬同城化发展，落实宁镇扬综合交通、产业发展、公共服务等领域一体化专项规划，加快宁句城际轨道建设，做好宁扬城际轨道前期工作。扎实做好援藏援疆、对口支援合作等各项工作。

（二）实施创新驱动发展战略，高标准建设创新名城。认真落实市委市政府“两个一号文件”，坚持“六化同步”，实施“六大计划”，推进创新名城建设迈出新步伐、实现新突破。

培育创新主体。实施科技型企业培育计划，建立高新技术企业梯度发展推进机制，净增高新技术企业900家、力争1000家，打造“科创企业森林”。抓住科创板推出的有利机遇，建立科创板上市企业梯队。发挥政府资金撬动作用，支持股权投资基金、保险资金、融资担保机构为创新创业提供更加精准的融资服务。深化培养、评价、流动、激励机制改革，更大力度支持企业引进高端人才。加快技术市场化进程，建设一批技术转移转化的专业机构、队伍和平台，构建专业化创新服务体系。

增强自主创新能力。实施综合性科学中心建设计划，推动网络通信与安全紫金山实验室高效运作，启动建设“扬子江生态文明创新中心”，加快取得一批重大原创性成果。聚焦产业前瞻领域，高水平启动一批重大科技专项，高标准建设一批重大研发平台，加强产业核心技术攻关，加快打造科技产业创新中心。实施新型研发机构提质计划，高质量发展新型研发机构，新增备案新型研发机构100家以上。

构建开放创新格局。实施全球创新资源集聚计划，自觉融入国际创新网络，精心举办南京创新周活动，加快建设海外协同创新中心、离岸科技企业孵化器等合作平台，招引一批高端项目、人才团队和科技服务机构，使南京直通全球创新最活跃的地区。坚持走融通发展之路，进一步推动校地、校企、校校、社企之间深度融合，探索建立产业协同创新学院，发展校友经济，推进军民融合协同创新，形成紧密协作的创新共同体。推动区域创新一体化，积极参与长三角城市群创新网络建设，高标准建设国家集成电路设计服务产业创新中心，打造苏南国家自主创新示范区“升级版”。

营造一流创新生态。实施创新载体升级计划，强化政府赋权、市场赋能，抓好“1+N”高新园区、环高校知识经济产业圈、城市“硅巷”等载体建设，推动江宁、仙林、江北三个大学城向创新城转变。实施法治服务环境提升计划，强化知识产权保护，高水平建设好江北新区法治园区、河西法律服务园，打造知识产权保护最严格城市。强化宽容失败的创新资本支持，提供宽容失败的创新金融服务，建立宽容失败的创新绩效评价，营造宽容失败的创新社会环境。

（三）推动制造业高质量发展，提升产业高度和竞争力。推动制造业发展地标化、制造业盈利模式服务化、生产型服务业高端化、市场主体多元化，构建自主可控的现代产业体系。

深化产业地标打造。围绕“4+4+1”主导产业方向，在重点实施新能源汽车、集成电路两个产业地标行动计划基础上，围绕人工智能、软件和信息服务、生物医药等具有区域影响力和发展潜力的产业，制定行动计划，落实推进举措，聚焦聚力打造3—5个“全省第一、全国前三、全球有影响力”的产业地标，不断提升产业高度和竞争力。

加快重大产业项目建设。制定工业设备投资专项奖补政策，激励市场主体加大工业投入力度，促进工业投资占固定资产投资比重快速提高。抓好重大招商活动签约项目落地转化，提升合同履约率、资金到账率、项目开工率。推动西门子数控研发及生产基地等一批优质项目开工建设，加快中兴通讯智能制造基地、LG三元锂电池、长安新能源汽车等一批百亿级先进制造业项目建设，推动FMC智能电动汽车等一批龙头项目顺利投产。抓好邦奇自动变速箱扩建等一批技改项目，重点支持企业智能化改造。

推动先进制造业与现代服务业深度融合。顺应制造业服务化发展趋势，推动生产型制造向服务型制造转变、以产品为中心向以用户为中心转变。大力发展工业互联网，加快培育研发设计、检测检验、营销培训等专业化服务企业，鼓励企业拓展总集成总承包、全生命周期管理、远程故障诊断及维护、网上支付结算等新业务，加速构筑智能化、绿色化、服务化的工业发展新体系。加强政策引导和扶持，实施一批示范项目，培育一批示范企业，净增规模以上工业企业200家。

推动传统产业转型升级。强化“以亩产论英雄”理念，推动低效园区、低效楼宇、低效用地等空间载体资源高效利用、高水平开发。运用好工业企业资源集约利用综合评价成果，实施差别化政策，引导资源要素向高效益、高产出、高技术、高成长性企业集聚。加快“两钢两化”产能转移规划方案制定步伐，启动梅钢传统产能转移和总部经济建设，推动“两钢两化”加快培育引进一批优质项目。推动江北新材料科技园打造一流的新材料产业集聚区。加快推进一批水泥企业矿山禁采、关停工作。

（四）深化改革开放，增强经济社会发展的动力活力。深化市场化改革，扩大高水平开放，促进经济行稳致远。

更大力度深化改革。深化国资国企改革，试点职业经理人制度，完成一批重点企业混合所有制改革。实施财政预算绩效管理改革，进一步提高政府财政资金使用效益。成立地方金融监管机构，积极争取创建国家金融科技示范区和绿色金融改革创新试验区。持续深化“放管服”改革，落实审批服务便民化要求，纵深推进工程建设项目审批制度改革试点，打响“864”改革品牌。统筹推进生态文明、社会事业、社会管理等领域改革。抓好江宁集成改革试点。抓好国家信用示范城市建设等一批试点任务，打造一批具有标志性和引领性的改革新亮点。

加快提升对外开放水平。加强同“一带一路”沿线国家和地区的产能合作与文化经贸往来，加大市场开拓力度，推动外贸进出口稳步增长、结构持续优化。加大国家服务贸易创新发展试点力度，深入开展国家跨境电子商务综合试验区建设，大力发展外贸综合服务平台等外贸新模式。更大力度复制推广自贸区政策举措，争取设立中国（江苏）自由贸易试验区南京片区。加快空港、江海港、高铁港三大枢纽经济区建设，力争南京空港、江北海港设立保税物流中心，创建国家临空经济示范区。深化宁台、宁港、宁新合作，办好两岸企业家紫金山峰会。

扎实开展“招商突破年”活动。强化“市外就是外”理念，坚持国资、民资、外资三资齐上，以先进制造业招商和科技资源集聚为重点，推动优质项目加快汇聚。建立完善招商大数据平台，办好金洽会、软博会、世界智能制造大会等重大活动，突出定点招商、精准招商，开展产业链招商、精细化招商，提升招商有效性。强化江北新区、11个经开区和15个高新区招商主阵地、主力军作用，强化抓项目导向，落实超常规举措，跑出新一轮高质量招商加速度。

推动民营经济健康发展。坚持“两个毫不动摇”，落实支持民营经济健康发展30条。推动民营企业纾困与发展基金实质性运作，用好民营企业转贷互助基金，做强市级政策性融资担保公司。落实国家降税、退税、减免税等政策，降低企业用电用工用地等各类要素成本。全面实施市场准入负面清单制度，放宽市场准入，加大政府采购支持民营企业力度，推动民营企业建设创新创业载体，提高民资在混合所有制企业中的比重。

打造全国最优营商环境示范城市。全面落实优化营商环境100条，坚持自我革新、刀刃向内、精准发力，持续打造营商环境“升级版”。大力推进“互联网+政务服务”，建立全市政务服务大数据库，推进政府数据向社会开放，打造企业服务总门户。进一步优化提升高铁、机场换乘服务体系，推动禄口机场打造全国服务最佳机场。

（五）提升城市功能品质，加快建设“美丽古都”。遵循城市发展规律，顺应群众品质需求，提升城市规划建设管理水平。

高水平规划城市。围绕“创新名城、美丽古都”愿景，深化城市总体规划修编，启动国土空间规划编制，优化控制性详细规划，更大力度推动“多规合一”，提升规划的系统性、国际性和标识性，引领南京发展迈向“扬子江时代”。持续推进“城市双修”、城市设计，塑造城市特色，彰显古都风貌。

高品质建设城市。进一步完善东山、仙林副城功能，加快河西新城、南部新城以及溧水、高淳副城建设发展，抓好鼓楼滨江、铁北地区、雨花两桥等片区更新改造，推进老城南更新与保护。大力推进长江五桥、和燕路、建宁西路等5条过江通道建设，加快推进锦文路、龙潭等过江通道前期工作，续建6条、开工6条总长237公里地铁线路。深入实施城市交通堵点治理，建成光华路东延工程，力争开工机场二通道绕城北段、惠民大道综合改造等一批重点工程。加快推进省园艺博览会博览园项目。整治提升夫子庙等12个片区环境，综合整治湖南路等20条主次干道、93个主城老旧小区、116条背街小巷。

高标准治理城市。实施城市绿化、彩化、亮化工程，提升新街口、鼓楼等城市核心区景观照明品质，打造外秦淮河、玄武湖等城市亮化视觉走廊。推进与新加坡智慧城市合作试点工作，打造东部地区信息数据中心，建设全国一流新型智慧城市。实施交通堵点治理三年行动计划，着力治理行车堵、停车难问题，营造更加畅通的出行环境。整体推进垃圾分类，建成江北废弃物综合处置中心一期工程，提高餐厨垃圾处理率和建筑垃圾资源化利用率，抓好全域“公厕革命”、共享单车管理。

（六）打好三大攻坚战，为决胜全面小康打下坚实基础。聚力打好防范化解重大风险、精准脱贫、污染防治三大攻坚战重点战役，使城市发展更加和谐、更具活力、更可持续。

狠抓长江大保护。坚持“共抓大保护、不搞大开发”战略导向，把南京长江两岸打造成为绿色生态带、转型发展带、人文景观带和严管示范带。强化生态红线刚性管控，制定长江岸线保护详细规划，全面加强长江岸线管理。实施生态保护修复工程，强化重点行业污染整治，严格防控船舶污染，加快船舶洗舱水处置能力建设，推进长江南京段生态保护和修复取得实质性进展。

加强生态环境治理。推进中央环保督查“回头看”和突出环境问题整改。大力推动钢铁、化工、水泥等非电行业减煤，强化扬尘管控和尾气污染治理，开展重点行业企业挥发性有机物治理，改善大气环境质量。持续巩固黑臭水体整治成果，全面开展劣V类水体整治，实施91个劣V类水体水环境提升工程，加快排口整治和活水保质工程建设，推进铁北、城南等污水处理厂扩建、改造；加快污水收集主次干管建设，推进金川河、外秦淮河等流域地下管网普查修复，基本完成雨污分流攻坚任务，实现北十里长沟、金川河、外秦淮河等入江支流断面水质持续达标。完善污染地块名录及开发利用负面清单，做好土壤及固废污染防治，完成矿山治理200万平方米。

加快推进脱贫攻坚。深入推进脱贫致富奔小康行动，确保已建档立卡的贫困人口脱贫销号、所有贫困村脱贫出列。加快提升经济薄弱村发展能力，确保全市所有建档立卡低收入人口家庭人均可支配收入达到9000元，所有经济薄弱村（欠发达村）年稳定收入达到100万元。完成商洛市、西宁市脱贫帮扶任务。

着力防范重大风险。深入推进防范和处置非法集资专项行动，扎实开展互联网金融和交易场所专项整治，密切关注、稳妥化解重点企业资金链风险。进一步加强政府债务管理，严格控制债务增量，严格按计划化解政府性债务，积极有效防范债务风险。坚持“房住不炒”定位，完善房地产调控长效机制，促进全市房地产市场平稳健康发展。

（七）加快江北新区建设，全力打造江北新主城。把江北新区发展放到更加突出的位置，高点定位、提速发展。

深化“两城一中心”建设。以台积电项目为龙头，加快集聚集成电路上下游企业，全力打造全国集成电路产业新高地。围绕基因检测、精准医疗、创新药物研发等重点方向，加快要素集聚，加强与国际顶尖研究机构合作，争取国家和省支持，力争国际健康城“三中心一高地”建设取得新突破。加快打造新区创业创新基金小镇，推动一批现代金融项目落地入驻，形成新金融业态集聚效应。

提升新区综合承载能力。以新区核心区为重点，高质量推进智慧城市、地下空间、综合管廊等重大项目建设，深入开展环境综合整治和景观提升，加快重点片区建设。全力推动新区快速路网、轨道交通、市政道路建设，构建新区立体交通体系。加强学校、医院等公共服务设施和能力建设，加快江北图书馆新馆、南京美术馆新馆建设，确保南京一中高中部、南丁格尔护理学院、南医大四附院等一批民生项目如期竣工，建成新区新市民中心。

加快新区改革开放步伐。推动新区与浦口、六合两区联动协作，统筹推进直管区、共建区、协调区融合发展。加快行政审批“信息化、智能化、标准化”建设，探索建立与新区发展相适应的街道管理体制机制，构建新型基层综合行政执法体制。放大服务贸易创新发展试点成果，提升新区开放型经济发展水平。

（八）实施乡村振兴战略，推进农业农村现代化。制定出台乡村振兴战略规划，实施现代农业提质增效等“十项工程”，走出一条具有南京特色的特大城市乡村振兴道路。

推进城乡高质量融合发展。有序推进农村土地制度改革，扎实做好利用集体建设用地建设租赁住房试点。深化农村集体产权制度改革，筹建南京农村产权交易中心。统筹优化城乡建设、产业集聚、生活居住等空间布局，完成450公里农村公路提档升级、新改建43座农路桥梁，提升“四好农村路”建管养护水平，推动优质教育、卫生资源向农村延伸。

加快农业现代化建设步伐。推进农业全产业链开发和一二三产深度融合，加快发展创意休闲农业、旅游观光农业和农村电子商务等新业态。高水平建设南京国家现代农业产业科创园，推动41个重点农业园区整合优势、提质发展，全年建设高标准农田7万亩、设施农业1.5万亩、农业机械化水平达87%。高标准建设白马国家农业高新技术产业示范区，探索现代水乡农业升级版路径。积极培育新型农业经营主体，促进农民合作社、家庭农场规范发展，推进农业产业化联合体建设。坚持质量兴农、绿色兴农，确保绿色优质农产品比重达40%，农产品质量安全总体合格率稳定在98%以上。抓好“米袋子”“菜篮子”工程，为市民群众提供更多的优质供给。

打造升级版都市美丽乡村。更高水平推进“千村示范、万村整治”工程，做好垃圾分类处理、污水治理、厕所革命和村容村貌提升工作，农村生活垃圾分类处理行政村基本全覆盖，全市规划布点村污水处理设施覆盖率达到90%以上，完成650公里农村道路绿化彩化建设。深化农村殡葬改革，推进移风易俗，进一步提高乡村文明水平。大力推进6个田园综合体建设，高标准打造乡村旅游精品线路和重点项目。全年培育省级特色田园乡村10个，建设美丽乡村示范村80个、宜居村100个。

（九）做强文化特色和优势，提升城市软实力。坚持文化自信，彰显文化魅力，以文化涵养城市精神、塑造城市品牌，增强城市创造力、吸引力。

打造文化高地。大力培育和践行社会主义核心价值观，深化文明城市创建。加快新媒体发展速度，推进传统媒体和新兴媒体融合发展。传承和弘扬中华优秀传统文化，加强文化遗产保护传承利用，加快中华门历史文化片区建设，实施南京名人馆、金陵刻经处博物馆等项目，推动十竹斋笺谱复刻等文化复兴工程，推进中国明清城墙、海上丝绸之路联合申遗。

推进“文化+”融合发展。大力推进“文化+旅游”，全面推动国家全域旅游示范城市创建工作，加快实施金陵小镇、华侨城文化旅游综合体、南捕厅历史文化街区等重大文化旅游项目。大力推进“文化+科技”，办好中国（南京）文化科技融合成果交易会、南京创意设计周等活动，加快建设一批文化和科技融合产业示范基地。大力推动“文化+体育”，创建全民运动健身模范市，办好2019年篮球世界杯，筹备好2020年室内田径世锦赛。大力发展文化事业，深入实施文化惠民“百千万”工程。

扩大城市影响力。开展全球城市营销，讲好南京故事、传播南京声音，提升南京的国际知名度和美誉度。进一步放大历史文化名城品牌，办好世界知名城市“南京周”“一带一路”国际文学论坛、媒体合作论坛等系列活动，推动文化“走出去”。加强对外友好交流，进一步深化青年文化交流和国际友城交往。

（十）保障和改善民生，增进民生福祉。把人民对美好生活的向往作为奋斗目标，多办民生实事，增进民生福祉。

扎实做好就业工作。聚焦高校毕业生、农村富余劳动力等重点群体，加大就业帮扶和技能培训力度，稳住就业基本盘，全年城镇新增就业24万人。突出以创业带动增收，持续提升南京“双创”强磁场，吸引各类人才创业圆梦。拓展居民增收渠道，实施差别化收入分配激励政策，完善职工工资正常增长机制，拓宽居民经营性收入、财产性收入渠道，推动知识、技能等要素价值在收入分配中得到更加合理体现。

提升社会保障水平。稳妥推进社保费征管职责划转，深入实施全民参保计划，推动实现法定人群全面覆盖、待遇水平合理增长。完善城镇职工、城乡居民基本养老保险制度，扩大企业年金覆盖面。优化全民医保制度体系，实施全市统一的城乡居民医保和大病保险制度，启动长期护理保险制度建设，深化医保支付方式改革。落实好社会救助、困境留守儿童关爱保护等兜底保障措施，探索多渠道促进社会力量参与“大救助”工作。完善住房保障机制，加大公租房货币化补贴、高校毕业生租房补贴等措施落实力度。新开工保障房450万平方米、竣工200万平方米。实施棚户区改造300万平方米。改善农村贫困家庭住房条件。

推进教育优质均衡发展。优化教育规划布局，鼓励优质资源向新区、郊区、保障房片区辐射，加强薄弱地区教育资源配置。继续推动学前教育优质普惠发展、义务教育学校标准化建设和普通高中布局优化三项工程，加快职业教育高质量发展。建立校外培训机构长效管理机制，促进民办教育健康发展。进一步完善“弹性离校”等惠民举措。

加快健康南京建设。推动医疗卫生资源优化布局、增加供给，开工建设市妇幼保健院丁家庄新院区。加快临床重点专科建设，争创国家临床医学研究中心。深化公立医院改革，加大医疗、医保、医药联动改革力度。提升农村基层社区卫生服务能力和水平，做好迎接国家卫生城市复审工作。支持溧水区打造“健康中国”样本。推进养老机构标准化建设，支持社会运营养老机构发展。

深化社会治理创新。深入推进创新网格化社会治理机制工作，大力开展全国社区治理和服务创新实验区工作，抓好社区专职工作者队伍建设。深化国防动员和双拥工作，做好退役军人事务工作，争创国家双拥模范城市“九连冠”。加强食品药品安全管理，完善应急管理体制，严格落实安全生产责任制。强化平安南京、法治南京建设，加强信访维稳工作，健全完善社会治安防控体系，深入推进扫黑除恶专项斗争，守住安全稳定底线。

各位代表：今年，我们将紧紧围绕广大市民最关心、关注的问题，全力办好交通出行、养老服务、学前教育、生态环境、便民服务、住房改善等十类28件覆盖面广、受益面大、与群众生活密切相关、获得感强的民生实事，切实增进民生福祉。同时，我们还将集中资源、聚焦重点，围绕市委市政府支持江北新区和各区发展的“十二项重要事项”，逐一制定具体方案并扎实推进实施，打造独特品牌优势，营造浓厚发展氛围。

三、全面加强政府自身建设

打铁还需自身硬。新的一年，我们要把党的领导贯穿到政府工作的各个方面，牢固树立“四个意识”，始终坚定“四个自信”，坚决做到“两个维护”，在思想上政治上行动上始终同以习近平同志为核心的党中央保持高度一致，以永不懈怠的精神状态和一往无前的奋斗姿态，践行新思想、承担新使命、展现新作为，努力打造人民满意的服务型政府。

一是坚持对标找差，鼓励创新创造，努力形成“更担当”的实干作风。把解放思想作为干事创业的逻辑起点，牢固树立不甘平庸、志存高远、永争一流、追求卓越的发展导向，着力营造勇于创新、善于创造、敢于突破的工作氛围，扛起南京责任，体现南京担当，勇立潮头、奋勇搏击，奋力推动各项工作走在全国全省前列。持续推进对标找差创新实干推动高质量发展活动，对标先进城市找准短板，聚焦重点领域逐项突破，树起南京高度，展现南京风貌，凝心聚力、克难奋进，推动各项工作再上新台阶。认真落实“三个区分开来”要求，用好鼓励激励、容错纠错、能上能下“三项机制”，坚持严管与厚爱结合、激励与约束并重，为担当者担当、为负责者负责、为干事者撑腰，形成以担当为荣、以发展为重、以奋斗为乐的发展环境。

二是坚持用户思维，注重客户体验，努力锻造“更优质”的政府服务。坚持问题导向，转换工作视角，把“用户思维、客户体验”理念融入政府治理和服务全链条，更多地从群众和企业角度去想问题、办事情、定政策、抓落实，把麻烦留给政府，把方便送给企业，把评判交给群众，不断提高市民对政府服务的感受度和体验度。着力构建“亲”“清”新型政商关系，建立完善与企业定期沟通互动、企业家座谈会、领导干部挂钩联系企业等机制，定期开展“支持实体经济发展主题服务月”活动，切实做到“四个主动”。统筹推进政府机构改革，进一步优化政府机构设置和职能配置，着力提升政府治理能力现代化水平。开展“十四五”规划编制前期研究工作。深入推进法治政府建设，严格按法定权限、法定程序行使权力，坚持以法治思维、法治方式推动工作，着力营造更加优良的法治环境。

三是坚持勤政廉政，全面从严治党，努力营造“更清朗”的政治生态。严格落实中央八项规定及其实施细则精神，严防“四风”问题反弹回潮。把“表态多调门高、行动少落实差”等突出问题作为治理重点，严肃整治形式主义、官僚主义新表现。统筹规范督查检查考核工作，不简单以留痕多少评判工作好坏，更注重考核细则化繁就简，营造考核注重实绩、工作重在平时导向，不搞花拳绣腿，不要繁文缛节，不做表面文章，为基层和干部“松绑减负”。认真贯彻国务院“约法三章”要求，严控“三公”经费增长。完善科学民主决策机制，自觉接受人大法律监督、政协民主监督，加强行政监督和审计监督。高标准高质量抓好中央巡视反馈意见整改，严格落实意识形态工作责任制，坚决消除“杨、季”等腐败案件恶劣影响。切实履行党风廉政建设主体责任，坚持无禁区、全覆盖、零容忍，把反腐的岗哨钉在工程建设、征地拆迁、扶贫攻坚、惠民利民等重点领域上，真正做到清清白白做人、干干净净做事，以实际行动向党和人民交出高质量的全新答卷。

各位代表：大道至简、实干为要。让我们更加紧密团结在以习近平同志为核心的党中央周围，在省委省政府和市委的坚强领导下，不忘初心、牢记使命，锐意进取、担当作为，以“创新名城、美丽古都”建设的崭新业绩，向新中国成立70周年献礼！
